# Genome Sequence of *Aeromicrobium erythreum* NRRL B-3381, an Erythromycin-Producing Bacterium of the *Nocardioidaceae*

**DOI:** 10.1128/genomeA.00300-16

**Published:** 2016-04-21

**Authors:** Erin A. Harrell, Eric S. Miller

**Affiliations:** Department of Plant & Microbial Biology, North Carolina State University, Raleigh, North Carolina, USA

## Abstract

*Aeromicrobium erythreum* NRRL B-3381 has a 3,629,239-bp circular genome that has 72% G+C content. There are at least 3,121 coding sequences (CDSs), two rRNA gene operons, and 47 tRNAs. The genome and erythromycin (*ery*) biosynthetic gene sequences provide resources for metabolic and combinatorial engineering of polyketides.

## GENOME ANNOUNCEMENT

*Arthrobacter* sp. strain NRRL B-3381 was part of a 1970 U.S. patent issued for an erythromycin process (1). Unlike other erythromycin processes, the NRRL B-3381 strain (isolated from Lajas Valley, Cabo Rojo, Puerto Rico) was notable for producing only erythromycin A and not the related compounds erythromycin B and C. From a large collection of industrially relevant actinobacteria, Sydney Brenner, then of the Medical Research Council (MRC) Molecular Genetics Unit, initiated a research program to genetically manipulate this nonfilamentous bacterium for polyketide combinatorial chemistry (2, 3). Since then, strain NRRL B-3381 has been taxonomically reclassified as the type genus and species *Aeromicrobium erythreum* (4), methods of plasmid transformation and gene disruption were developed (3, 5), and cosmid clones of *ery* (erythromycin) genes were isolated and sequenced (6). Although interesting metabolic manipulations of *A. erythreum* have been performed (7, 8), extensive uses of its polyketide synthase and other erythromycin biosynthesis genes have not been reported. Access to the complete genome sequence of the NRRL B-3381 strain may facilitate macrolide antibiotic development and other biotechnological uses of this and related *Actinobacteria* (9).

Total DNA was prepared using the Qiagen Gentra Puregene yeast/bact kit with overnight cultures of *A. erythreum* (collection strain designated AR18) grown at 30°C in 2xYT medium with shaking. Ten micrograms of purified DNA was processed with the Pacific Biosciences (PacBio) 10-kbp library kit in the NC State University Genomic Sciences Laboratory and then analyzed by single-molecule real-time (SMRT) RS II sequencing. Genome assembly was done with PacBio SMRT HGAP 2/Quiver using 75,640 reads with *N*_50_ of 5,716 bases (mean, 3,737 bases; 0.83 quality) totaling 282,723,636 bases. A total of 70,568 reads were mapped at 62.4× coverage to an assembled contig of 3,629,239 bases. No plasmid DNA was assembled. The genome is circular (by read overlap observations and PCR confirmation), has 72% G+C content, and is oriented in the GenBank file starting at 103 bases preceding the *dnaA* coding sequence (CDS).

The 3.6-Mbp genome was annotated with the NCBI Prokaryotic Genome Annotation Pipeline (10), with some manual curation. There are 3,121 CDSs, 270 pseudogenes needing further analysis, two rRNA operons (16S, 23S, and 5S), at least 47 tRNAs, and one potential transfer-messenger RNA (tmRNA). Methylation kinetics revealed m6A (N^6^-methyladenine), primarily at CTCCAG and CTGGAG (a *Bpm*I-like site).

Sequences of the erythromycin-related genes, including those encoding the methyltransferase resistance enzyme (*ermR*) and the 65-kbp *ery* gene cluster, are essentially as previously reported (2, 6) (accession no. AY623658). The *ery* gene cluster includes three polyketide (6-desoxyerythronolide B) synthase modules (*eryAI—AIII*) and the methyltransferases, dehydratase, isomerase, sugar transferases, etc. leading to erythromycin A. *Aeromicrobium marinum* DSM 15272 (accession no. CM001024) encodes the most proteins currently orthologous to proteins of *A. erythreum*, and orthologs are also found from *Nocardioides simplex* VKM Ac-2033D (11).

The available *A. erythreum* NRRL B-3381 genome sequence should provide a resource for comparative and evolutionary genomics and, as suggested by Sydney Brenner 30 years ago, facilitate metabolic and combinatorial engineering of polyketide biosynthesis in a genetically tractable unicellular actinobacterium.

### Nucleotide sequence accession number.

The genome sequence has been deposited in GenBank with accession no. CP011502. The version described in this paper is the first version.
